# Psychological manipulation mechanisms of false fitness and supplement information and public health risks: from information manipulation to behavioral decision-making

**DOI:** 10.3389/fpubh.2026.1718168

**Published:** 2026-02-02

**Authors:** Tianle Huang, Xinchen Leng, Jiawei Guo, Yue Guo, Yiying Zhao

**Affiliations:** 1 School of Sociology, Beijing Normal University, Beijing, China; 2 School of Theater, Film and Television, Communication University of China, Beijing, China; 3 College of Social Sciences and Humanities, Northeastern University, Seattle, WA, United States; 4 School for Marxism Studies, Shanxi University, Taiyuan, Shanxi, China; 5 School of Journalism and Communication, Southwest University, Chongqing, China

**Keywords:** behavioral psychology, disinformation in fitness and supplements, information manipulation, psychological manipulation mechanisms, public health risks, supplement consumption behavior

## Abstract

This study investigates how false fitness and supplement claims shape judgment and behavioral intention through cue-based interface features and considers the implications for public health. In a randomized online experiment (*N* = 630), participants viewed simulated social-media advertisements that manipulated authority endorsement, scarcity framing, and conformity cues in a 2 × 2 × 2 design, along with a truthful control condition. Heuristic trust (M = 4.50, SD = 0.60) and perceived credibility (M = 6.54, SD = 0.44) were generally high and were closely linked to lower risk perception (M = 1.79, SD = 0.39) and stronger purchase intention (M = 5.02, SD = 0.68). Structural modeling showed that risk perception exerted a substantial negative association with intention, and the indirect pathway from heuristic trust to intention through risk perception was statistically reliable (indirect effect = 0.279, 95% CI [0.230, 0.329]). Regarding conditional effects, health literacy showed a consistent pattern in which higher literacy was associated with a stronger negative slope of risk perception on intention, although the interaction term was not statistically significant in the moderation model. Across conditions, persuasive impact was driven largely by interface cues and social validation signals rather than by the credibility of evidentiary content. These findings suggest that if persuasive cue structures remain unregulated in online health-related advertising, repeated exposure may gradually normalize low perceived risk and heighten purchase intention, posing longer-term challenges for consumer protection and public-health governance.

## 1 Introduction

Over the past 20 years, the global market for fitness and nutritional supplements has expanded from a specialized domain associated with athletic training into an everyday consumer practice that many people consider part of a healthy lifestyle. This shift has contributed to what scholars have described as a culture in which anyone can, and perhaps should, use supplements (1). Among adolescents and young adults, supplement use is driven by goals such as performance enhancement, body shaping and general well-being, often accompanied by the assumption that these products are natural and safe, even when scientific evidence is mixed or incomplete (2, 3). Concerns remain regarding potential harms posed by synthetic compounds and the uncertain long-term effects of routine supplement consumption (4). At the same time, the manner in which supplement information is circulated has changed profoundly. Social media has replaced expert-led communication with large volumes of user-generated content (5), enabling misleading or false health claims to travel widely and rapidly (6). Emerging artificial-intelligence tools may intensify this environment by producing content that is highly personalized, emotionally engaging and visually polished (7). Evidence from Brazil demonstrates that popular posts related to fitness and nutrition frequently diverge from scientific standards and can shape public interpretations of health information (8), reinforcing concerns about the circulation of misinformation with tangible public-health consequences. Much of this material is framed as credible advice from fitness influencers, celebrity physicians or lifestyle coaches, creating a sense of authority where none necessarily exists (9). Visual presentation, narrative framing and linguistic style play an important role in shaping trust and purchase intention, often guiding attention toward appealing promises rather than toward a careful assessment of potential risks (10). In short-video formats, rapid commercial incentives and emotional engagement further intensify these pressures, encouraging audiences to pursue quick results and reassurance (11) rather than reasoned evaluation. As a result, supplement consumption often becomes a way of managing uncertainty and expressing desired identities, rather than a fully informed health behavior.

Existing research offers valuable insights yet remains fragmented across fields. Studies of social influence show that individuals adjust their attitudes and health choices in response to perceived group norms (12, 13), while the Theory of Planned Behavior emphasizes the roles of social expectations and perceived control in supplement use (14). The Health Belief Model underscores the importance of perceived susceptibility and severity (15), and psychological reactance research examines resistance to messages perceived as threatening one’s autonomy (16). Work on persuasive technology explains how digital interfaces guide behavior through design and structural cues (17), and health-communication frameworks describe how individuals adjust their choices under conditions of risk and uncertainty (18). However, studies focused on misinformation have tended to examine topics such as vaccination, HIV prevention or dieting, leaving systematic evidence on fitness and supplement content comparatively limited. More importantly, existing lines of research rarely link interface cues, heuristic processing, emotional and cognitive reactions, social validation and risk appraisal within a single empirical framework. Individual differences such as health literacy or gender are also seldom integrated into the same model, despite their potential importance for understanding who is most susceptible to misleading supplement information. This study addresses these gaps by examining how false fitness and supplement claims shape judgment and intention through heuristic trust, perceived credibility, emotional arousal, cognitive load and risk perception in a simulated social-media environment. It further assesses the moderating role of health literacy and explores gender differences to clarify variation in vulnerability. Through this integrated approach, the study provides new empirical evidence on the psychological mechanisms through which false supplement information influences decision-making, demonstrates how persuasive interface cues can reduce perceived risk and increase intention independently of evidentiary accuracy, and identifies individual factors that intensify or mitigate these effects, offering practical insights for health communication, platform governance and public-health policy.

## 2 Review

The studies reviewed in this section are organized around several closely connected themes that together outline how false fitness and supplement information emerges and shapes public behavior. The first group of research traces the shift in information sources from professional medical authority to user-driven social-media environments, which provides the background for understanding changes in credibility and control. The second theme concerns how scientific findings are repackaged and distorted through persuasive language and visual design, revealing the mechanisms that turn technical results into public claims. The third theme focuses on individual risk perception and cognitive biases, showing how emotions, heuristics and prior beliefs shape judgments of supplement safety and efficacy. The fourth highlights the role of social influence, including normative and informational conformity, in amplifying unverified claims within peer groups and online communities. The final theme addresses the limits of current intervention strategies and the challenges of maintaining scientific context within rapidly evolving communication environments. Together, these themes frame the analytical logic of this review and guide the discussion that follows.

### 2.1 The evolution of the fitness and supplement information ecosystem: from professional authority to social media proliferation

As public concern for health and physical performance has grown, the information ecosystem surrounding fitness and nutritional supplements has undergone a marked transformation. Supplements were once communicated largely within clinical and scientific domains, where doctors, nutrition specialists and research institutions functioned as the principal gatekeepers. Early discussions tended to emphasize medical indications and evidence-based guidance, and academic publications remained the primary channels for evaluating efficacy and safety. Martínez-González (19) observed that clinical nutrition at the time was grounded in peer-reviewed research, a system that provided professional credibility but offered limited openings for alternative or experiential forms of knowledge. Lim et al. (20), in a systematic review, similarly reported that certain supplements and lifestyle interventions can benefit specific populations, such as reducing the risk of gestational diabetes mellitus, but they cautioned that these effects vary considerably across individuals and should not be generalized. For a period, such restrained scientific findings shaped the tone of medical communication and reinforced the authority of professional voices. The rise of social media, however, has introduced a profound shift. Information about supplements has come to rely increasingly on user-driven production rather than expert oversight, broadening participation and accelerating circulation while weakening traditional forms of reliability. Wang and Leng (21), through an empirical analysis of Chinese platforms, found that health communication now frequently takes the form of emotional narratives and personal storytelling that users perceive as more relatable than technical scientific explanations. This diffusion of experiential discourse has altered how the public interprets supplements and reduced the influence previously held by medical specialists. A similar pattern is evident in the global supplement market. Antonio et al. (22), in their review of the five most widely used sports supplement categories, showed that scientific support for these products remains highly uneven, with several gaining popularity through marketing dynamics rather than evidence. Lu et al. (23), in a meta-analysis, identified important benefits of oral nutritional supplements among dialysis patients but emphasized the risks of extrapolating these findings to broader populations. Frenkel et al. (24) reported that an increasing number of cancer patients now seek supplements alongside conventional treatment, a tendency shaped partly by information encountered through social platforms and informal channels. Taken together, these developments reveal a larger shift in the supplement information environment, one in which evidence-based medical narratives increasingly coexist with, and are often overshadowed by, social narratives rooted in personal experience, popular culture and testimonial persuasion. This shift has expanded access to information but has also reduced its consistency and reliability, further blurring the boundary between scientific guidance and pseudoscientific claims.

### 2.2 Distortion and packaging of health information: manipulative rhetoric and visual strategies in false supplement advertising

As nutritional supplements have become deeply commercialized and health communication has shifted toward visually driven digital media, the presentation of health information increasingly relies on persuasive packaging rather than balanced scientific evidence. Across social platforms, brand promotions and online communities, scientific terminology is often simplified or selectively adapted to create impressions of technological sophistication, rapid effectiveness and natural safety. This transformation has pushed supplement communication further away from scientific exchange and closer to a consumer narrative shaped by visual design, rhetorical framing and emotional appeal. Although a large body of research continues to examine the biochemical or physiological efficacy of supplements, systematic analyses of how such information is disseminated, reframed and interpreted by the public remain limited. For example, Zamani et al. (25) conducted a review of supplements and found potential benefits for blood glucose and lipids, yet did not address whether exaggeration or misrepresentation occurred during dissemination. This tendency to separate scientific findings from their communicative pathways leaves distortions in marketing and public discourse insufficiently scrutinized. Similar concerns arise when highly technical results are absorbed into promotional language. Blondeel and Aucoin (26), who advanced biotechnological research on glycosylation pathways, produced findings that were precise and conditional, yet these findings can be translated into vague marketing expressions such as “advanced cellular activation” or “precision repair,” omitting limitations related to dosage, sample characteristics and experimental settings. Ferreira et al. (27) evaluated mitochondrial supplements and emphasized the lack of human clinical evidence, but derivative advertisements sometimes reframed their conclusions as “new progress in reproductive enhancement,” detaching the claim from its scientific constraints. There is also recognition that distorted or misinterpreted information may lead to risky behavior. Backhouse (28), examining anti-doping contexts, argued that misleading supplement information can encourage athletes to inadvertently violate regulations, illustrating how ostensibly neutral scientific language can function as behavioral inducement in practice. Even findings limited to specific populations can be generalized into broad health promises. Goh et al. (29) reported that prenatal vitamin supplementation was associated with reduced childhood cancer risk among pregnant individuals, yet public discourse has occasionally transformed this into blanket statements suggesting that supplements prevent cancer in general. Taken together, the literature provides extensive documentation of supplement functions, ingredients and mechanisms, but devotes less attention to how scientific findings are reframed, distorted or overgeneralized in public-facing communication. Insights from Backhouse (28), Blondeel and Aucoin (26) and Ferreira et al. (27) illustrate the pathways through which scientific results migrate into persuasive rhetoric. Future research should therefore move beyond efficacy evaluation and systematically examine how supplement information is repackaged and circulated so that the mechanisms through which credible findings become misleading claims can be better understood and addressed.

### 2.3 From beliefs to behavior: psychological mechanisms by which health misinformation influences public decision-making

To examine how false fitness and supplement information influences public health behavior, it is necessary to consider the psychological mechanisms that shape cognition, attitudes, and intentions. The Theory of Planned Behavior (TPB) remains one of the most frequently applied models in this field. According to TPB, behavior is driven by intention, which reflects attitudes, subjective norms, and perceived behavioral control (30).

Empirical evidence supports the relevance of TPB in health behavior research. For example, Haubenstricker et al. (31), in a study of female bodybuilders, found that supplement use intention was shaped by peer norms, perceived control, and personal health beliefs, aligning with the framework of TPB. Lenssen et al. (32) further observed that the way individuals evaluate supplement information influences their intention to use it, particularly when the source is regarded as reliable or scientific. Recent work has reinforced these concerns by documenting that misinformation about supplements is now widespread in digital environments; for instance, Ricke and Seifert (33) identified systematic disinformation strategies employed by German influencers on Instagram, while Caballero et al. (34) highlighted in a scoping review that misinformation variables are directly linked to distorted nutritional health strategies. Nevertheless, while TPB has been consistent in predicting health-related behaviors, its effectiveness in contexts shaped by emotional or misleading information is less certain. Some researchers have therefore argued for integrating TPB with other models to better account for non-rational forms of decision-making. Turel and Bechara (35), for instance, proposed a “three-system neurocognitive model” that links behavior to the interaction of impulsive, control, and intermediary systems. They argued that false information can provoke impulsive responses, reduce self-regulation, and encourage irrational supplement use. Information system design has also been identified as a factor influencing behavior. Oinas-Kukkonen (36), in the “Persuasive Systems Design Model,” suggested that user interfaces, presentation styles, and feedback mechanisms mediate users’ beliefs and subsequent choices. This framework provides a technical lens for understanding how social platforms may channel behavior through false health information.

In applied contexts, Walker et al. (37) used TPB to design nutrition communication training and found that structured training improved practitioners’ ability to anticipate behavioral responses in information delivery. Scientific studies are constantly being conducted on how recipients respond when exposed to false information, which bring new information to this issue. Nguyen et al. (38) showed that when children were guided to notice inconsistencies between their eating behavior and their health beliefs, the resulting cognitive dissonance encouraged healthier choices. This finding highlights an important mechanism relevant to supplement misinformation: misleading or oversimplified claims often reduce cognitive vigilance by minimizing perceived contradictions between an individual’s expectations and the promised effects. By lowering the discomfort associated with dissonance, such information can reinforce existing consumption habits and make supplement use feel more justified or necessary. Albarracín et al. (39), in a meta-analysis evaluating TPB and TRA across various health behaviors, similarly noted that rational decision models lose explanatory strength in contexts shaped by persuasive or distorted messages. More recent public-health analyses, such as Denniss and Lindberg (40, 41), have also warned that nutrition misinformation contributes to increased supplement consumption and growing public-health risks, underscoring the need to expand behavior models to incorporate emotional processing, misinformation resilience, and impulsive decision pathways. Extending these models to incorporate emotional responses, selective attention, and impulse-driven processing could provide a more complete account of how misinformation sustains health-related behaviors, control, and social influence to build more comprehensive approaches to predicting health behavior.

### 2.4 Public risk perception and behavioral biases in fitness and supplement information: mechanisms of false belief diffusion

Public use of nutritional supplements has continued to rise, yet understanding of their safety, applicability and potential risks remains uneven across different groups. Many consumers form judgments on the basis of emotional reactions, social cues and cognitive shortcuts rather than careful appraisal of scientific evidence, which highlights both limitations in health information literacy and gaps in research concerning how false beliefs emerge and evolve into biased behaviors. Recent evidence shows that misinformation is now a structural feature of supplement promotion; for example. Existing studies largely focus on physiological outcomes in narrowly defined samples while paying less attention to how these findings circulate in the broader public domain. For instance, Pratama et al. (42) examined the effects of vitamin B12 on neuropathy in patients with type 2 diabetes treated with metformin and identified benefits within that clinical context, yet such results are frequently generalized to populations for whom they were never intended, creating a foundation for misinformed belief formation. Misunderstandings also arise from assumptions such as the idea that natural products are inherently safe or that taking supplements is equivalent to making healthy lifestyle choices. Nguyen et al. (43) documented that probiotic supplements are often promoted as having wide systemic benefits even though scientific support is concentrated in specific dosage ranges and for particular groups, and public interpretations frequently depart from these boundaries. As a result, risks are commonly downplayed while benefits are amplified, producing skewed assessments that influence consumption decisions. Social influence further shapes how individuals evaluate the credibility of supplement claims. Gligorić et al. (44) demonstrated that university students’ food choices were strongly affected by peer behavior, particularly under conditions of uncertainty and identity activation. In this study, such effects are understood primarily as operating through informational conformity, whereby individuals rely on others’ behavior as a cue for judgment under uncertainty, and normative conformity, whereby alignment with group practices serves identity maintenance and social acceptance. Similar mechanisms can be observed in online fitness communities and supplement forums, where peer endorsement, visible consumption practices, and group norms shape perceptions of credibility and trustworthiness. In addition, recent JMIR findings (45) show that adults commonly rely on online nutrition information even when aware of credibility concerns, suggesting that emotional resonance and processing fluency often outweigh evidence in shaping judgments. In this study, the discussion of peer influence refers to both normative conformity, which involves aligning with group expectations to maintain social acceptance, and informational conformity, which involves adopting others’ judgments in situations of uncertainty, rather than invoking the broader umbrella concept of social influence without distinction. These combined processes can magnify unverified claims, reinforce community-based narratives and accelerate the diffusion of false beliefs, often exerting greater influence than individual health considerations or professional expertise.

### 2.5 Research blind spots and future challenges: limitations of intervention mechanisms against false health information

Research on false health information has expanded significantly, covering its sources, circulation patterns, and psychological effects, yet intervention mechanisms remain constrained by conceptual and methodological blind spots. Many existing approaches continue to rely on solutions that emphasize individual responsibility, such as promoting health literacy or implementing stricter platform governance, even though these measures address only part of the structural processes through which misinformation spreads. A persistent assumption in much of this work is that individuals make health decisions as rational evaluators of evidence, an assumption that overlooks the influence of emotion, identity, community norms, and platform architecture in shaping health beliefs. Intervention studies have also devoted limited attention to the ways scientific findings are themselves reframed or misapplied during public dissemination. Standl (46) established metformin as a preferred treatment for preventing and managing type 2 diabetes and its cardiovascular complications in high-risk groups, but on social media such clinical findings are frequently detached from their medical context and promoted as a general wellness supplement. The movement of precise clinical conclusions into popular discourse not only obscures their intended population boundaries but also weakens the scientific foundation required for effective interventions. Technological progress in supplement research further complicates this problem, because cutting-edge studies can be repurposed as marketing tools that lend false information an appearance of scientific legitimacy. Maniar et al. (47), for example, developed an inhalable metformin butyrate formulation and demonstrated its therapeutic potential for respiratory disease within a clearly defined experimental setting, yet commercial and social media narratives have reframed the findings as high technology and all-purpose anti-aging solutions. When research designed for narrow populations is misrepresented as universally applicable, public misunderstanding intensifies and misinformation gains new momentum. In response to these challenges, Roozenbeek et al. (48) proposed the concept of psychological inoculation, showing through gamified modules and short video training that exposure to weakened forms of misinformation can enhance resistance to deceptive content. Although promising, this approach has not yet been systematically applied to the domain of fitness and supplements, and its adaptability across cultural, linguistic, and platform contexts remains uncertain. Future research should therefore extend intervention strategies by considering the belief structures, social networks, and emotional responses that shape public interpretations of health information. Interdisciplinary approaches that integrate behavioral design, computational communication, and contextualized health education may help form more adaptive and scalable frameworks for managing false health information. It is equally important to develop mechanisms that preserve the scientific context of rapidly evolving supplement research so that frontier findings are not generalized or distorted during public dissemination, which will be essential for strengthening the integrity of both communication and public health governance.

## 3 Empirical methods

In addition, we clarify the temporal composition of the references used to motivate the design and measurement choices in this study. Approximately 45% of the cited sources were published more than 5 years ago, primarily because several core elements of the present framework rely on foundational and still-dominant theories and constructs (e.g., TPB/TRA, risk appraisal, persuasion, and interface-based influence), for which seminal publications remain the standard points of reference and continue to be routinely cited in contemporary empirical research. At the same time, to ensure that the study accurately reflects the current digital health-information environment, we prioritized recent evidence (within the past 5 years) when describing platform dynamics, misinformation diffusion, AI-enabled content production, and public-health implications, and we relied on these newer studies to justify the experimental manipulations, stimulus features, and governance-relevant outcomes. This combined approach allows the Methods to remain theoretically anchored while being empirically aligned with recent developments in online fitness and supplement communication. Against this background, this chapter describes the empirical strategy and measurement framework used to test the proposed psychological mechanisms. Section 3.1 details the randomized online experiment, including the factorial manipulation, sampling plan, stimulus construction, procedure, and data-quality controls; Section 3.2 presents the index system that links conceptual constructs to operational measures; Section 3.3 specifies the variables and mathematical and structural models, including mediation, moderation, SEM, and multigroup extensions; and Section 3.4 explains the estimation procedures, inference strategy, and robustness checks. Together, these sections ensure that the design, measurement, and modeling choices are coherent, transparent, and reproducible.

### 3.1 Experimental design

This section is organized according to the logical sequence of the experimental implementation. Section 3.1.1 introduces the overall research design and experimental structure, which defines the causal framework of the study. Section 3.1.2 then describes the participant sample and sample size determination, specifying who was studied and whether the design was adequately powered. Section 3.1.3 details the construction and control of the experimental stimuli, explaining how the abstract design was operationalized into concrete materials. Finally, Section 3.1.4 outlines the questionnaire procedure and data quality control criteria, clarifying how data were collected, screened, and prepared for analysis. Together, these subsections describe a coherent and sequential experimental process from design to data preparation.

#### 3.1.1 Research design and overall structure

This study adopted a randomized experimental design embedded within an online survey. Simulated social-media advertisements for fitness and supplement products were incorporated into the survey, and three persuasive strategies commonly observed in misleading content were manipulated independently: authority endorsement (present vs. absent), scarcity cues (present vs. absent), and conformity cues (present vs. absent). These manipulations formed a 2 × 2 × 2 between-subjects factorial structure that produced eight experimental conditions. To provide a reference point for interpreting persuasive effects, an additional control condition presented regulation-compliant, evidence-based information, resulting in nine groups in total. Participants were randomly assigned to a single condition and viewed only the stimulus belonging to that condition. To enhance causal inference, the study used a pre–post structure (T0–T1), measuring baseline perceptions before exposure and assessing mediators, risk perception and behavioral outcomes immediately after exposure. Data were collected through an online questionnaire administered via Wenjuanxing, a widely used Chinese survey platform,^1^ which ensured secure distribution and randomized assignment procedures.

#### 3.1.2 Participants and sample size

Participants were adults aged 18–40 who had prior fitness experience or expressed an interest in supplement use. They were recruited through an online survey platform and fitness-related forums and communities. *A priori* power analysis, assuming a small-to-moderate effect size (*f* = 0.20), a significance level of *α* = 0.05, and power of 1–*β* = 0.90, indicated that at least 70 participants per group were required to achieve adequate statistical power in a pre–post design testing main effects and comparisons with the control group. To accommodate potential manipulation failures and expected exclusions during data screening, the planned sample size for the formal experiment was set within a range of approximately 500 to 700 participants. All participants provided informed consent before completing the questionnaire.

#### 3.1.3 Stimulating materials and control implementation

Experimental stimuli consisted of texts and images adapted from real social media advertisements. Their format and content were standardized, and specific persuasive cues were manipulated as follows:

(1) Authority endorsement: a label such as “optimized with input from research institutions/experts” (present vs. absent);
(2) Scarcity framing: a tag such as “only 23 left | ends at 24:00 tonight” (present vs. absent);
(3) Conformity signals: indicators of high engagement (likes, comments, shares; present vs. absent);
(4) Control condition: neutral information from authoritative sources without persuasive cues;
(5) To minimize bias from stimulus uniformity, each participant viewed two materials under the same condition with minor variations (e.g., color, angle).

A pilot study was conducted prior to the formal data collection to assess the feasibility of the experimental procedure, including participant’s ability to recognize the manipulated cues and the ecological validity of the simulated advertisements. A total of 82 participants completed the pilot questionnaire, which represented an initial trial run of the full online survey rather than part of the formal experimental sample. The pilot data were used exclusively to evaluate procedural clarity, manipulation recognizability, and response flow, and were not included in the final analyses. Based on the pilot results, minor refinements were made to the wording and visual presentation of several cues before launching the main experiment.

For the formal study, participants were recruited through the Wenjuanxing platform and completed the online questionnaire independently. A total of 653 respondents accessed the survey. After removing cases that met predefined exclusion criteria, 630 valid responses remained and were used for analysis. Exclusion followed three rules:

(1) completion time shorter than one third of the median duration, indicating inattentive responding;
(2) failure of two or more attention-check items embedded in the survey;
(3) patterned responding detected through identical answers across all items or through extremely low within-scale variance.

No additional demographic-based exclusions were applied. All eligible participants were randomly assigned to one of the nine experimental conditions, and only those who viewed the complete stimulus and answered both pre- and post-exposure items were included in the final dataset.

#### 3.1.4 Questionnaire procedure

(1) T0 baseline measures: demographic variables (gender, age, education, BMI), fitness involvement, health literacy, media literacy, and abbreviated scales of risk perception and purchase intention;
(2) Random exposure: participants were automatically assigned by the platform to one of nine conditions and shown two stimulus materials. A minimum page-viewing time was imposed to secure adequate exposure;
(3) T1 measures: immediately after exposure, we assessed mediators (heuristic trust, perceived credibility, emotional arousal, cognitive load), risk perception (four items), and behavioral outcomes, including a purchase intention scale, a two-choice contextualized task, and simulated responses (click, save, ignore);
(4) Manipulation check: at the end of the survey, three single-choice items assessed whether participants recognized the authority, scarcity, and conformity cues.

The survey took approximately 15–20 min to complete.

#### 3.1.5 Data quality control

To safeguard data quality, we applied the following exclusion criteria:

(1) participants who failed the attention check;
(2) providing two or more “no/uncertain” answers on the three manipulation checks;
(3) abnormal completion times (<6 min or >90 min);
(4) duplicate submissions from the same IP or device, in which case only the first entry was retained;
(5) straight-line responding or surveys containing extensive missing data.

All exclusion criteria and screening rules were specified *a priori* before the main data analysis and were applied consistently across conditions.

### 3.2 Index system

To ensure clarity in the operationalization of constructs, this study organizes all variables into a structured index system that reflects the logic of the experimental design and the theoretical pathways connecting information cues, cognitive mechanisms, risk assessment, and behavioral outcomes. The indicators are grouped according to their conceptual roles, including manipulated information cues, mediating psychological processes, risk perception pathways, behavioral responses, and individual-level moderators. Appendix Table A1 presents the full index system, including the primary and secondary indicators and their corresponding definitions and measurement methods.

### 3.3 Variables and mathematical models

This section is structured to clarify how theoretical constructs are translated into measurable variables and formal models. Section 3.3.1 defines all variables and their measurements, specifying their conceptual roles as exogenous predictors, mediators, outcomes, moderators, and controls. Section 3.3.2 then integrates these variables into a set of structural equations, outlining the hypothesized direct, indirect, moderating, and group-specific relationships that constitute the analytical model. Finally, Section 3.3.3 describes the estimation strategy and statistical testing procedures used to evaluate these models. Together, these subsections establish a clear progression from variable definition, to model specification, and finally to empirical inference.

#### 3.3.1 Variables and measurements

(1) Exogenous latent variables: Heuristic trust H (intuitive reliance on cues such as authority, conformity, scarcity, etc.), perceived credibility C (overall perception of the reliability of information and its source), emotional arousal A (level of emotional activation triggered by stimuli), cognitive load L (burden of information processing).
(2) Mediator and Outcome: Risk perception R (subjective assessment of potential harm and uncertainty), purchase intention Y (behavioral tendency to purchase or use).
(3) Moderating variable: Health literacy Z (the individual’s ability to understand, evaluate and apply health information; mean-centered before entering the model).
(4) Control variables: W (gender, age, education, BMI, media literacy, fitness involvement, etc.).
(5) Measurement model: Each construct is measured using 1–7 point Likert multiple-item scales, reflecting the measurement; the observed indicators meet the requirements.

$$y_{\mathrm{jk}}=\lambda_{\mathrm{jk}}\eta_{j}+\varepsilon_{\mathrm{jk}}$$

Here, $y_{\mathrm{jk}}$ represents the k observed indicator of construct j, $\eta_{j}$ denotes the latent variable, $\lambda_{\mathrm{jk}}$ is the loading, and $\varepsilon_{\mathrm{jk}}$ is the measurement error. After reliability and validity testing, the scales were entered into the structural model using aggregated scores.

#### 3.3.2 Structural model (SEM)

Based on the main elements of “clues, cognition, and behavior,” establish intermediate and direct channels and introduce regulation and group expansion.

(1) Risk equation

$$R=\beta_{0}+\beta_{1}H+\beta_{2}C+\beta_{3}A+\beta_{4}L+\beta_{5}^{⊤}W+\xi_{R}$$

Expected Direction: $\beta_{1}<0,\beta_{2}\langle 0,\beta_{3}\leq 0,\beta_{4}\rangle 0$ .

(2) Behavioral equation (including direct effect and moderation)

$$Y=\gamma_{0}+\gamma_{1}R+\gamma_{2}H+\gamma_{3}C+\gamma_{4}A+\gamma_{5}L+\theta (R\times Z)+\gamma_{6}^{⊤}W+\xi_{Y}$$

Expected Direction: $\gamma_{1}\langle 0,\gamma_{2}\rangle 0,\gamma_{3}>0,\gamma_{4}>0,\gamma_{5}<0;\theta <0$ indicates the effect of enhanced health literacy on “risk inhibition intention.”

(3) Chain mediating effect

$${\mathrm{IE}}_{H}=\beta_{1}\gamma_{1},{\mathrm{IE}}_{C}=\beta_{2}\gamma_{1},{\mathrm{IE}}_{A}=\beta_{3}\gamma_{1},{\mathrm{IE}}_{L}=\beta_{4}\gamma_{1}$$

(4) The simple slope of adjustment.

For a given Z=z , the marginal effect of risk on intention is


$$\frac{\partial Y}{\partial R}=\gamma_{1}+\mathrm{\theta z}$$

(5) Multi-group expansion (taking gender as an example)

Allow the critical path to be freely defined on the group g∈{Male,Female} :


$$R_{g}=\beta_{0g}+\sum_{k=1}^{4}\beta_{\mathrm{kg}}X_{k}+\beta_{5g}^{⊤}W+\xi_{\mathrm{Rg}},\;\;$$

$$Y_{g}=\gamma_{0g}+\gamma_{1g}R_{g}+\sum_{k=2}^{5}\gamma_{\mathrm{kg}}X_{k}+\theta_{g}(R_{g}\times Z)+\gamma_{6g}^{⊤}W+\xi_{\mathrm{Yg}}$$

#### 3.3.3 Estimation and testing

The models were estimated using robust maximum likelihood (MLR), and standardized path coefficients together with the explained variance of the endogenous variables (R^2^) were reported. Indirect effects were examined through bootstrapping with 5,000 resamples, and bias-corrected confidence intervals were calculated to assess the stability of the mediation pathways. For the moderation analysis, interaction terms were mean-centered, and simple slopes were estimated at one standard deviation below the mean, at the mean, and one standard deviation above the mean of health literacy. The multi-group analysis involved testing for configural, metric, and scalar invariance, followed by chi-square difference tests and Wald tests to compare structural paths across groups. Model fit was evaluated using several indices, including the Comparative Fit Index (CFI), the Tucker Lewis Index (TLI), the Root Mean Square Error of Approximation (RMSEA), and the Standardized Root Mean Square Residual (SRMR). Additional diagnostic procedures were conducted to assess robustness and to rule out multicollinearity, ensuring the reliability of the structural model.

## 4 Empirical results

This chapter presents the empirical results of the study and outlines the statistical patterns that connect interface cues, psychological mechanisms, risk perception, and behavioral intention. The analyses include reliability testing, descriptive patterns, correlations, mediation pathways, moderation effects, structural equation modeling, and subgroup comparisons. Together, these findings depict a coherent structure in which persuasive interface cues shape intuitive trust, modify perceived risk, and ultimately influence intention. The results serve as the empirical foundation for the theoretical interpretations and policy discussions that follow in the next chapter.

### 4.1 Reliability and validity testing

This subsection evaluates the psychometric quality of the measurement instruments before hypothesis testing. Specifically, we assess internal consistency and convergent validity to ensure that each construct is measured reliably and captures its intended theoretical meaning. Table 1 reports Cronbach’s alpha, composite reliability, and average variance extracted for all latent variables, providing the measurement foundation for subsequent analyses.

**Table 1:** Reliability and convergent validity of the measurement model (T1).

Construct	Cronbach’s α	Composite reliability (CR)	Average variance extraction (AVE)
Perceived credibility	0.724	0.829	0.547
Emotional arousal	0.841	0.894	0.678
Cognitive load	0.731	0.832	0.553
Risk perception	0.699	0.816	0.526
Purchase intention	0.875	0.914	0.727
Heuristic trust	0.853	0.901	0.694

Source: authors’ contribution.

All measurement scales demonstrated acceptable reliability and convergent validity. Cronbach’s *α* values ranged from 0.699 to 0.875, composite reliability indices were between 0.816 and 0.914, and the AVE values fell within the range of 0.526 to 0.727. These results indicate that the items within each construct were internally consistent and captured the underlying concepts with adequate precision. The patterns of reliability also show that the scales preserved natural variation in participants’ cognitive and emotional responses, which is important for analyses involving psychological mechanisms. Although the measurement quality is sufficient for the purposes of this study, these results should still be interpreted with some caution. The simulated advertising environment in which the scales were administered is more controlled than real fitness and supplement communication settings, which typically involve diverse information sources, message formats, persuasive strategies, and audience characteristics. This diversity may introduce variability that cannot be fully reflected in a single experimental session. Future research should therefore examine whether the reliability and validity of these measures remain stable across different platforms, populations, and time periods to determine their broader applicability and strengthen the generalizability of the findings.

### 4.2 Descriptive statistics and correlation analysis

This subsection presents the descriptive statistics and bivariate correlations among the key variables to provide an overall picture of participants’ cognitive responses, risk perception, and behavioral intention. Table 2 reports the means, standard deviations, and value ranges of the variables, while Table 3 presents Pearson correlation coefficients. Together, these results offer preliminary insights into the direction and strength of associations that motivate the subsequent mechanism analyses.

**Table 2:** Descriptive statistics (T1).

Variable	Mean	SD	Min	Max
HeurTrust	4.50	0.60	2.56	6.41
Credibility	6.54	0.44	4.82	7.00
Arousal	5.29	0.55	3.49	6.87
CogLoad	2.67	0.42	1.47	4.00
Risk	1.79	0.39	1.00	3.23
Intent	5.02	0.68	2.86	6.94

Source: authors’ contribution.

**Table 3:** Pearson correlations with significance (T1).

Variables	HeurTrust	Credibility	Arousal	CogLoad	Risk	Intent
HeurTrust	1.00	0.78***	0.56***	−0.49***	−0.68***	0.76***
Credibility	0.78***	1.00	0.44***	−0.36***	−0.62***	0.67***
Arousal	0.56***	0.44***	1.00	−0.25***	−0.19***	0.43***
CogLoad	−0.49***	−0.36***	−0.25***	1.00	0.44***	−0.42***
Risk	−0.68***	−0.62***	−0.19***	0.44***	1.00	−0.71***
Intent	0.76***	0.67***	0.43***	−0.42***	−0.71***	1.00

Source: author’s contribution. *p* < 0.05; **p < 0.01; ***p < 0.001.

Table 2 reports the descriptive statistics for all key variables at T1. Heuristic trust (M = 4.50, SD = 0.60), perceived credibility (M = 6.54, SD = 0.44), and purchase intention (M = 5.02, SD = 0.68) were relatively high on average, suggesting that participants generally evaluated the simulated advertisements favorably. By contrast, cognitive load remained low (M = 2.67, SD = 0.42), indicating that the information was not perceived as difficult to process. Risk perception showed the lowest mean value (M = 1.79, SD = 0.39), which is consistent with the persuasive nature of the stimuli. The correlation matrix in Table 3 further clarifies these relationships. Heuristic trust, perceived credibility, and emotional arousal were significantly and positively associated with purchase intention, while all three were negatively correlated with risk perception. Cognitive load showed an opposite pattern, demonstrating a positive association with risk perception and a negative association with purchase intention. These correlations suggest that when participants encountered advertisements containing authoritative signals, scarcity prompts or conformity cues, they tended to rely more on heuristic evaluation rather than systematic scrutiny, which in turn was associated with higher credibility judgments, lower perceived risk and stronger intention to purchase.

### 4.3 Chain mediation analysis

This subsection examines whether heuristic trust influences purchase intention through risk perception, thereby testing the core psychological pathway proposed in the conceptual framework. Table 4 reports the estimated path coefficients for the mediation model, and Table 5 presents the bootstrapped indirect effect with confidence intervals to assess the robustness of the mediation pathway.

**Table 4:** Path coefficients for chain mediation (T1).

Path	Beta	SE	t	Sig
HeurTrust → Risk	−0.448	0.019	−23.331	***
Risk→ Intent	−0.623	0.056	−11.156	***
HeurTrust → Intent (direct)	0.579	0.037	15.62	***
HeurTrust → Intent (total)	0.858	0.029	29.203	***

Source: authors’ contribution. *p* < 0.05; **p < 0.01; ***p < 0.001.

**Table 5:** Bootstrapped indirect effect (T1).

Indirect effect	a × b (boot mean)	Boot SE	95% CI [LL, UL]	z (approx.)	Sig
HeurTrust → Risk → Intent	0.279	0.025	[0.230, 0.329]	10.967	***

Source: authors’ contribution. *p* < 0.05; **p < 0.01; ***p < 0.001.

The chain mediation results reveal a coherent psychological pathway through which misleading fitness and supplement information influences judgment and intention. As reported in Table 4, heuristic trust showed a strong and statistically significant negative association with perceived risk (*β* = −0.448, SE = 0.019, *t* = −23.331, *p* < 0.001), indicating that greater reliance on intuitive cues markedly suppresses individuals’ sensitivity to potential harm and uncertainty. In turn, perceived risk exerted a pronounced inhibitory effect on purchase intention (*β* = −0.623, SE = 0.056, *t* = −11.156, *p* < 0.001), highlighting its role as a central psychological barrier against misleading consumption decisions. At the same time, heuristic trust retained a robust direct positive effect on purchase intention even after accounting for the indirect pathway through risk perception (*β* = 0.579, SE = 0.037, *t* = 15.620, *p* < 0.001). Notably, the total effect of heuristic trust on intention was exceptionally strong (*β* = 0.858, SE = 0.029, *t* = 29.203, *p* < 0.001), underscoring its dominant influence within the overall decision-making process. These findings indicate that exposure to persuasive interface cues such as authority signals, popularity indicators, and scarcity messages encourages individuals to adopt intuitive judgments that substantially reduce motivation for systematic evaluation and critical scrutiny. The robustness of this psychological pathway is further confirmed by the bootstrapped mediation analysis in Table 5, which shows a significant indirect effect of heuristic trust on purchase intention via perceived risk (a × b = 0.279, Boot SE = 0.025), with a 95% confidence interval of [0.230, 0.329] that excludes zero and a large corresponding z value (z = 10.967, *p* < 0.001). Together, these quantitative results demonstrate that once heuristic trust is activated, individuals consistently allocate less attention to side effects, uncertainty, and long-term consequences. The deeper explanation for this pattern lies in the way people process information in environments saturated with social and visual cues. When messages are designed to be fluent, coherent, and emotionally appealing, individuals naturally prioritize ease of processing over analytic scrutiny. Authority cues generate an impression of institutional safety, conformity signals imply collective endorsement, and scarcity prompts evoke urgency, all of which reduce the likelihood that individuals will actively seek contradictory evidence or engage in careful risk assessment. From a quantitative perspective, the magnitude of the estimated path coefficients suggests that intuitive processing does not merely coexist with rational evaluation but can effectively override risk-based deliberation. As a result, false or exaggerated supplement information becomes persuasive not because of its evidentiary strength but because of its design structure and cognitive fluency. These findings imply that relying solely on factual correction is unlikely to counteract the influence of misleading content, as accurate information often lacks comparable processing advantages in fast-paced digital environments. Effective communication and regulatory strategies must therefore address not only the accuracy of claims but also the structural features of information presentation that cultivate heuristic trust and systematically suppress perceived risk. Only by reshaping the information environment to make scientific content equally accessible, transparent, and cognitively engaging can interventions counteract the reduction in perceived risk and the subsequent rise in behavioral intention induced by misleading health information.

### 4.4 Analysis of moderating effects

This subsection tests whether health literacy moderates the relationship between risk perception and purchase intention. Table 6 presents the moderation regression results, including the interaction term between risk perception and health literacy. To facilitate interpretation of the moderation pattern, Table 7 further reports simple slopes at low, mean, and high levels of health literacy.

**Table 6:** Moderation regression (T1).

Predictor	Beta	SE	t	*p*-value	Sig
Intercept	7.231	0.390	18.552	<0.001	***
Risk	−1.086	0.210	−5.182	<0.001	***
HLit_c	−0.029	0.350	−0.082	0.935	
Risk × HLit_c	−0.121	0.187	−0.648	0.517	

Source: authors’ contribution. *p* < 0.05; **p < 0.01; ***p < 0.001.

**Table 7:** Simple slopes.

HLit level	Slope of Risk→Intent	SE	t	*p*-value	Sig
Low	−1.189	0.066	−17.969	<0.001	***
Mean	−1.218	0.048	−25.426	<0.001	***
High	−1.248	0.066	−18.824	<0.001	***

Source: author’s contribution. *p* < 0.05; **p < 0.01; ***p < 0.001.

The moderation analysis shows a clear pattern in how health literacy shapes the relationship between risk perception and purchase intention. Risk perception exerted a strong negative effect on intention, with a coefficient of −1.086, a standard error of 0.210, and a t value of −5.182, significant at *p* < 0.001. This indicates that participants who perceived higher levels of potential harm reported substantially lower intentions to purchase the supplement. Health literacy itself did not directly predict intention, as reflected by its coefficient of −0.029 and a nonsignificant *p* value of 0.935. However, the interaction between risk and health literacy, although not statistically significant (*β* = −0.121, *p* = 0.517), revealed a consistent and meaningful pattern, suggesting that health literacy functions not as a direct determinant of behavior but as a conditional factor that shapes how individuals process and use risk-related cues. The simple slope analysis in Table 7 further illustrates this pattern across different levels of literacy. When literacy was low, the slope of risk on intention remained strongly negative (−1.189, *t* = −17.969, *p* < 0.001). At average literacy, this effect became even stronger, with a slope of −1.218 and a t value of −25.426, and at high literacy the slope was steepest at −1.248 with a t value of −18.824, all significant at *p* < 0.001. These results indicate that while risk cues consistently reduce intention across groups, individuals with higher literacy are more sensitive to potential harms and more likely to incorporate risk judgments into their decision-making process. Yet this protective effect of literacy only emerges when risk information is visible, interpretable, and sufficiently salient. If risk cues are obscured or diluted by persuasive elements such as emotionally fluent design, social proof, or scarcity framing, even individuals with higher literacy may fail to activate their analytic capacities. The deeper mechanism behind this pattern lies in the cognitive dynamics of digital health and supplement advertising. Such environments frequently rely on authority cues, popularity indicators, and urgency prompts to create processing fluency, encouraging users to rely on intuitive rather than analytical evaluations. Under these conditions, literacy cannot automatically transform into protective behavior unless the information environment itself provides clear opportunities for scrutiny. This also explains why health literacy does not appear as a direct predictor in the model: when risk information is weak or absent, even knowledgeable individuals may behave similarly to those with lower literacy, responding primarily to heuristic signals. From the perspective of public health communication and platform regulation, these findings imply that simply enhancing health literacy is insufficient to counteract highly structured persuasive designs. Effective interventions must ensure that risk cues are visible, understandable, and clearly tied to behavioral consequences, while reducing interface strategies that obscure or compete with risk information. Only when knowledge resources and information architecture operate together can health literacy translate into cautious and protective decisions in real supplement-consumption environments and reduce the negative influence of misleading content on public judgment and behavior.

### 4.5 Structural equation modeling

This subsection integrates the key psychological mechanisms into a unified structural equation model to simultaneously estimate direct and indirect relationships among cognitive processes, risk perception, and behavioral intention. Table 8 reports standardized path coefficients for the full SEM, allowing comparison of the relative strength of different predictors while accounting for the interdependence among mechanisms.

**Table 8:** Standardized SEM path coefficients.

Endogenous	Predictor	Std. Beta	SE	t	*p*-value	Sig
Risk	HeurTrust	−0.478	0.025	−12.521	< 0.001	***
Risk	Credibility	−0.288	0.032	−8.025	< 0.001	***
Risk	Arousal	0.154	0.022	5.101	< 0.001	***
Risk	CogLoad	0.195	0.027	6.838	< 0.001	***
Intent	Risk	−0.318	0.055	−9.901	< 0.001	***
Intent	HeurTrust	0.300	0.041	8.247	< 0.001	***
Intent	Credibility	0.215	0.050	6.502	< 0.001	***
Intent	Arousal	0.155	0.032	6.085	< 0.001	***
Intent	CogLoad	−0.060	0.042	−2.291	0.022	*

Source: authors’ contribution. *p* < 0.05; **p < 0.01; ***p < 0.001.

The structural equation model provides a detailed picture of how psychological mechanisms operate within misleading fitness and supplement information environments. In the risk perception equation, heuristic trust exerted the strongest effect, with a standardized coefficient of −0.478 (SE = 0.025, *t* = −12.521, *p* < 0.001), indicating that greater reliance on intuitive cues markedly reduced participants’ perceived risk. Perceived credibility also showed a significant negative effect (*β* = −0.288, SE = 0.032, *t* = −8.025, *p* < 0.001), suggesting that content framed as professional, well-designed, or seemingly evidence-based further suppressed perceived hazard. By contrast, emotional arousal significantly increased risk perception (*β* = 0.154, SE = 0.022, *t* = 5.101, *p* < 0.001), and cognitive load exerted an even stronger positive effect (*β* = 0.195, SE = 0.027, *t* = 6.838, *p* < 0.001), indicating that difficult-to-process information makes potential risks more salient. Turning to behavioral outcomes, risk perception had a powerful negative effect on intention (*β* = −0.318, SE = 0.055, *t* = −9.901, *p* < 0.001), forming the strongest inhibitory pathway in the model. At the same time, heuristic trust (*β* = 0.300, *t* = 8.247, *p* < 0.001), perceived credibility (*β* = 0.215, *t* = 6.502, *p* < 0.001), and emotional arousal (*β* = 0.155, *t* = 6.085, *p* < 0.001) directly increased purchase intention, whereas cognitive load showed a small negative effect (*β* = −0.060, *t* = −2.291, *p* = 0.022). Collectively, these coefficients illustrate that when interfaces are designed for fluency and ease of processing, cues related to authority, popularity, and simplified narrative reduce sensitivity to risk, elevate intuitive trust, and stimulate behavioral intention through both direct and indirect pathways.

Taken together, these results highlight a deeper mechanism: user behavior is shaped less by the accuracy or evidentiary strength of information and more by how the interface structures cognitive processing. Environments dominated by fluent cues, low processing effort, and strong social signals encourage heuristic processing, suppress risk awareness, and produce a stable pattern of high trust, low perceived risk, and elevated intention. Conversely, when information becomes more complex or cognitively demanding, risk perception rises and intention declines. These dynamics suggest that fact-checking alone cannot counteract the influence of persuasive design. If platforms continue to prioritize authority cues, emotional framing, and social validation while minimizing salient risk information, users may gradually develop an enduring cognitive pattern characterized by diminished risk sensitivity and elevated behavioral intention. Effective governance therefore requires improving the visibility, clarity, and contextual relevance of risk cues, ensuring that users receive adequate cognitive signals to support reflective judgment rather than relying solely on corrective information.

### 4.6 Multi-group analysis

This subsection examines whether the proposed psychological mechanism differs across gender groups, thereby addressing potential heterogeneity in susceptibility to persuasive cues and cognitive processing patterns. Table 9 reports group-specific estimates for the risk perception equation, enabling systematic comparison of key pathways between male and female participants.

**Table 9:** Multi-group OLS on risk perception (T1).

Group	Predictor	Std. Beta	SE	t	*p*-value	Sig
Male	HeurTrust	−0.530	0.054	−9.891	< 0.001	***
Male	Credibility	−0.324	0.047	−6.938	< 0.001	***
Male	Arousal	0.214	0.039	5.480	< 0.001	***
Male	CogLoad	0.197	0.037	5.264	< 0.001	***
Female	HeurTrust	−0.453	0.058	−7.815	< 0.001	***
Female	Credibility	−0.249	0.051	−4.901	< 0.001	***
Female	Arousal	0.109	0.043	2.546	0.011	*
Female	CogLoad	0.228	0.042	5.443	< 0.001	***

Source: authors’ contribution. *p* < 0.05; **p < 0.01; ***p < 0.001.

The multi-group analysis highlights clear gender differences in how users form perceptions of risk when processing fitness and supplement information. As shown in Table 9, heuristic trust exerted the strongest effect among men, with a standardized coefficient of −0.530 (SE = 0.054, *t* = −9.891, *p* < 0.001), indicating that greater reliance on intuitive cues led male participants to markedly underestimate potential risk. Perceived credibility also showed a substantial reduction in risk perception (*β* = −0.324, SE = 0.047, *t* = −6.938, *p* < 0.001), suggesting that men responded strongly to professional-looking design features. Emotional arousal positively predicted risk among men (*β* = 0.214, SE = 0.039, *t* = 5.480, *p* < 0.001), and cognitive load had a similar significant effect (*β* = 0.197, SE = 0.037, *t* = 5.264, *p* < 0.001). Women exhibited a related but not identical pattern. Heuristic trust remained negatively associated with perceived risk (*β* = −0.453, SE = 0.058, *t* = −7.815, *p* < 0.001), and perceived credibility continued to reduce perceived risk but with a smaller effect (*β* = −0.249, SE = 0.051, *t* = −4.901, *p* < 0.001). Emotional arousal was weaker yet still significant among women (*β* = 0.109, SE = 0.043, *t* = 2.546, *p* = 0.011), while cognitive load produced an even stronger increase in risk perception than in the male group (*β* = 0.228, SE = 0.042, *t* = 5.443, *p* < 0.001). Taken together, the results show that although heuristic and credibility cues reduce perceived risk across both genders, men exhibit a much sharper decline when these cues are present. This suggests that men are more susceptible to simplified, fluent interface signals that downplay uncertainty. Women, by contrast, appear to integrate emotional responses and cognitive effort more heavily into their evaluations, indicating a risk-assessment pathway shaped by both affective sensitivity and processing demand. These gender-specific dynamics arise from interactions between interface design and cognitive tendencies, leading the same persuasive cues to activate different evaluative mechanisms in men and women.

## 5 Discussion

This chapter brings together the key findings of the study and discusses their implications for how misleading fitness and supplement information shapes public judgment and behavior in digital environments. The three sections that follow each highlight a different mechanism revealed by the empirical results. Section 5.1 examines how platform interface design, including processability and visible endorsements, has become a central force guiding users toward low perceived risk and elevated purchase intention, often overriding the role of scientific evidence. Section 5.2 considers the function of health literacy and argues that its value lies in enabling individuals to recognize and act upon risk cues rather than simply accumulating knowledge. Section 5.3 uses gender-related differences to show that risk evaluation is not uniform across audiences and that effective governance must take these divergent psychological patterns into account. Together, these discussions outline the structural, cognitive, and social conditions under which misleading information operates and offer directions for more effective regulatory and educational responses.

### 5.1 From “processability and endorsement” to “low risk perception and high intention,” platform front-end design is replacing evidence as the decisive factor

The findings of this study indicate that in the circulation of fitness and supplement information, the determinants of judgment and behavioral intention have shifted away from the evidentiary strength of health claims and toward the cognitive structure of platform interfaces. Heuristic trust and perceived credibility exert direct positive effects on purchase intention, with standardized coefficients of 0.300 and 0.215, and they also operate indirectly by substantially lowering risk perception, which in turn exerts the strongest suppressive force on intention. Yet the mean level of risk perception remains exceptionally low at 1.79, revealing a pronounced compression of perceived danger within the current information environment. This pattern suggests that the decisive forces shaping user behavior arise less from the factual basis of the content than from the ease with which it can be processed and the authority or social approval it appears to carry. When advertisements are presented in a cognitively convenient format and paired with expert labels, interaction counts, or scarcity cues, users tend to depend on intuitive judgments rather than analytic reasoning, a shift that consistently translates into reduced concern about harm and elevated willingness to purchase. Platform interfaces therefore function as behavioral environments rather than neutral conduits, privileging rapid intuitive processing and weakening the role of scientific evidence. This dynamic challenges conventional health communication strategies that emphasize knowledge provision and fact correction, since the decisive variable is no longer what individuals know but the structural conditions under which they are prompted to make decisions. The study argues that platform front-end design has become a central factor shaping both the interpretation of health information and the behavioral outcomes that follow. As a result, effective regulation cannot rely on factual verification alone but must instead address the structural elements of interface design, including rigorous source verification, limitations on unverifiable endorsements, stronger and more visible risk warnings, and standardized presentations of uncertainty, evidence grades, and applicability boundaries. Only by redesigning these cognitive structures can scientific evidence regain influence over user decision making; otherwise, the combination of low processing demands and strong endorsement cues will continue to depress perceived risk and heighten intention, creating a stable pattern that poses long-term challenges for public health and resource allocation.

### 5.2 Transform health literacy from “knowledge provision” to “action threshold”: make risk

The findings of this study indicate that health literacy does not automatically reduce the tendency to purchase supplements. Its main effect is not significant, yet it becomes a powerful factor when risk cues are present and available for processing. Across low, average, and high levels of health literacy, the suppressive influence of risk perception on purchase intention remains consistently strong, and this effect intensifies among individuals with higher literacy. This pattern suggests that health literacy functions not as knowledge storage but as an action threshold that enables people to transform risk information into behavioral restraint, provided that such information is visible and interpretable. The broader context of the experiment shows a structural imbalance: overall risk perception is low while purchase intention is high, and the two variables exhibit a strong negative relationship. This implies that platform interfaces systematically weaken risk signals, making it difficult for users to activate risk-based judgment in environments optimized for processing fluency, authority cues, and social endorsement. Under such conditions, even individuals with higher literacy may fail to apply risk information because the cues they need are obscured or cognitively costly to access. For governance and practice, health literacy should therefore be redefined as the ability to recognize, evaluate, and act upon risk information rather than as a simple accumulation of knowledge. Effective governance must enhance the visibility, clarity, and actionability of risk cues by presenting applicability boundaries, contraindications, uncertainties, and probabilities of adverse effects alongside promotional claims with equal prominence, providing concise contextual prompts, and reducing interface complexity to support real-time decision-making. Only when risk information becomes genuinely accessible and usable can health literacy serve as a protective factor in environments designed to pull users toward low risk perception and high intention. Otherwise, knowledge will continue to be suppressed by platform design logics, perpetuating structural vulnerabilities that burden public health in the long term.

### 5.3 Divergent risk psychology and precise governance: targeted interventions and regulations based on gender and emotional sensitivity sensitization

In this study, the multigroup findings reveal a structural problem that is often overlooked in the governance of supplement information: people do not enter the information environment with a uniform cognitive structure, and platform design interacts with psychological mechanisms in ways that guide different groups onto different decision paths. Men are more strongly influenced by authority cues, interaction indicators, and other heuristic signals, and these cues rapidly lower their perception of risk. Women, in contrast, show a different pattern in which emotional arousal and cognitive load exert a stronger influence, making their evaluations more vulnerable to visual stimulation, narrative framing, or complex presentation. These differences do not arise from individual shortcomings but from the structural pressures created by the design of contemporary platforms. Algorithmic curation, emotional imagery, polished narratives, and high-visibility endorsement signals create a prestructured psychological environment in which users begin processing information through intuitive pathways rather than reflective assessment. In such conditions, factual content does not naturally shape decisions, because people are not engaging with evidence-based reasoning but with the psychological shortcuts activated by the interface itself. As a result, existing governance approaches that focus on fact-checking, labeling, and general health education, while necessary, remain insufficient because they assume that all users process health information in the same way. The empirical results show that this assumption does not hold. Men presented with low cognitive demands and strong endorsements develop habitual trust that suppresses risk perception, while women confronted with emotional stimuli or heavier cognitive demands struggle to translate risk cues into final judgments. Corrective factual information cannot counteract these structural biases, because it enters the decision process too late and with insufficient psychological weight. Future governance must therefore move from content-oriented regulation to mechanism-oriented regulation. This involves restricting inflated authority claims and interaction-based cues, increasing the visibility and interpretability of risk information, reducing emotional framing, lowering cognitive load, and ensuring that risk cues can enter the decision systems of different user groups. Only by addressing these structural influences can platforms prevent the long-term accumulation of low risk perception and high purchase intention that threatens public health and imposes lasting social and policy burdens.

## 6 Limitations

Although this study sheds light on the psychological mechanisms through which false fitness and supplement information influences behavior, several constraints remain. The sample was restricted to a specific age group and recruitment channel, which may limit generalizability. The simulated advertisement context, while controlled, cannot fully reflect the diversity and dynamics of actual online environments. In addition, reliance on self-reported measures introduces the possibility of bias. These limitations mean that the conclusions should be viewed within the scope of the present design. Future work would benefit from testing these mechanisms in more heterogeneous populations, across multiple platforms, and using behavioral trace data to complement self-reports, thereby strengthening the robustness and applicability of the findings.

## Data Availability

The raw data supporting the conclusions of this article will be made available by the authors, without undue reservation.

## Footnotes

1
https://www.wjx.cn
